# Impression management in sex and gender neuroscience research reporting: the MAGIC guidelines

**DOI:** 10.1038/s41467-024-47261-0

**Published:** 2024-04-01

**Authors:** Gina Rippon, Katy Losse, Simon White

**Affiliations:** 1 Institute of Health and Neurodevelopment, School of Health and Life Sciences, Aston University, Birmingham, B4 7ET UK; 2 Independent Researcher, St Albans, UK; 3 MRC Biostatistics Unit, University of Cambridge, Cambridge, UK

**Keywords:** Publishing, Neuroscience

## Abstract

Here, the authors discuss guidelines to avoid miscommunication of findings in research into sex and gender-based differences in the brain.

## Teaser

Research into sex and gender-based differences in the brain is of key importance in many arenas. As findings will be scrutinised by both specialists and non-specialists, problems of miscommunication may arise. Guidelines to identify and avoid such problems are proposed.

## Background

Questions about the influence of sex and gender on the human brain have potentially widespread social and political significance, with the answers informing parenting and educational practices, shaping cultural norms and expectations, as well as determining individual actions such as career choice. References to research on sex and gender differences in the brain can be found informing diversity and inclusion initiatives, lessons in schools, and business approaches to leadership training, as well as in more populist genres, such as self-help and relationship advisory manuals^1–4^. This means that research reports on sex and gender and the brain are often the subject of widespread scrutiny, way beyond the research community that produced them, by enthusiastic and engaged but, possibly, non-expert audiences.

Accurate communication of any research findings is, of course, a core part of the scientific process, and is subject to many checks and balances within specialist publications. However, where a field is of widespread interest outside such publications, we consider it useful to have an extra requirement of ‘impression management’, with additional guidelines to ensure that the language used to describe findings to non-specialists is unambiguous and not open to misinterpretation or misunderstanding.

Specifically with respect to sex and gender in neuroscience research, it is particularly important that communication of research findings does not, for example, exaggerate the extent or importance of any differences found between women and men, or unjustifiably imply that such differences are fixed and inevitable, or that such differences might be interpreted as demonstrating inferiority in either group. Otherwise it can serve to sustain gender stereotypes, which can have damaging consequences. For example, the Fawcett’s Society’s Commission on Gender Stereotypes in Early Childhood has reported on the limiting effects of such stereotypes in education and employment^1^. Parliamentary enquiries into Equity in the Science and Technology workforce have found that gender stereotypes can contribute to lower take-up of STEM subjects among girls^5^. A belief in fixed, biologically-determined differences between females and males can undermine support for diversity and inclusion initiatives^6^.

As with any area of science, misunderstanding or misrepresentation of research findings can occur at any point along the chain of communication^7^. Publications related to sex and gender issues may attract the attention of general interest science outlets and/or mainstream media, due to public interest in such research. Social media may additionally circulate such reports; given the increased likelihood that the material is transmitted by non-experts, additional possibilities for distortion arise. The resulting over-simplified or even inaccurate statements may then appear in self-help books, training manuals, parenting guides etc. These can sustain inaccurate stereotypes about fixed or hardwired differences between males and females^1–4^.

But it should be acknowledged that their origins could be in original research paper themselves. There may be, for example, a disconnect between the actual numerical strength of the research findings and the inflationary language used to describe them in the narrative interpretation^8^. Similarly, failure to flag the influence of specific methodological choices can mislead as to the true significance (statistical and otherwise) of the findings^9^. The use of persuasive communication devices, such as mischaracterising the theoretical framework or the current state of knowledge in the area, can give a false impression of the reliability and consistency of relevant evidence bases^10^. A contemporary emphasis on the need for published work to have demonstrable impact beyond the immediate research arena can compound such problems.

There are, of course, existing safeguards in place within scientific research publishing practices to ensure methodological and analytical accuracy. Most important is the long-standing peer review process, with detailed editorial guidelines for reviewers, together with statistical controls such as CONSORT and STROBE, which focus attention on methodological precision and analytical accuracy^11,12^. Within the sphere of sex and gender research, the Sex and Gender Equity in Research (SAGER) Guidelines, for example, provide detailed checklists for ensuring that sex and gender variables are fully and accurately incorporated at each level of research design, analysis and interpretation^13^. The Open Science movement is operationalising safety checks across the research design board, including questionable research practices to which sex and gender research can be particularly prone, such as post-hoc analyses and over-generalisation^14^.

**Fig. 1:**
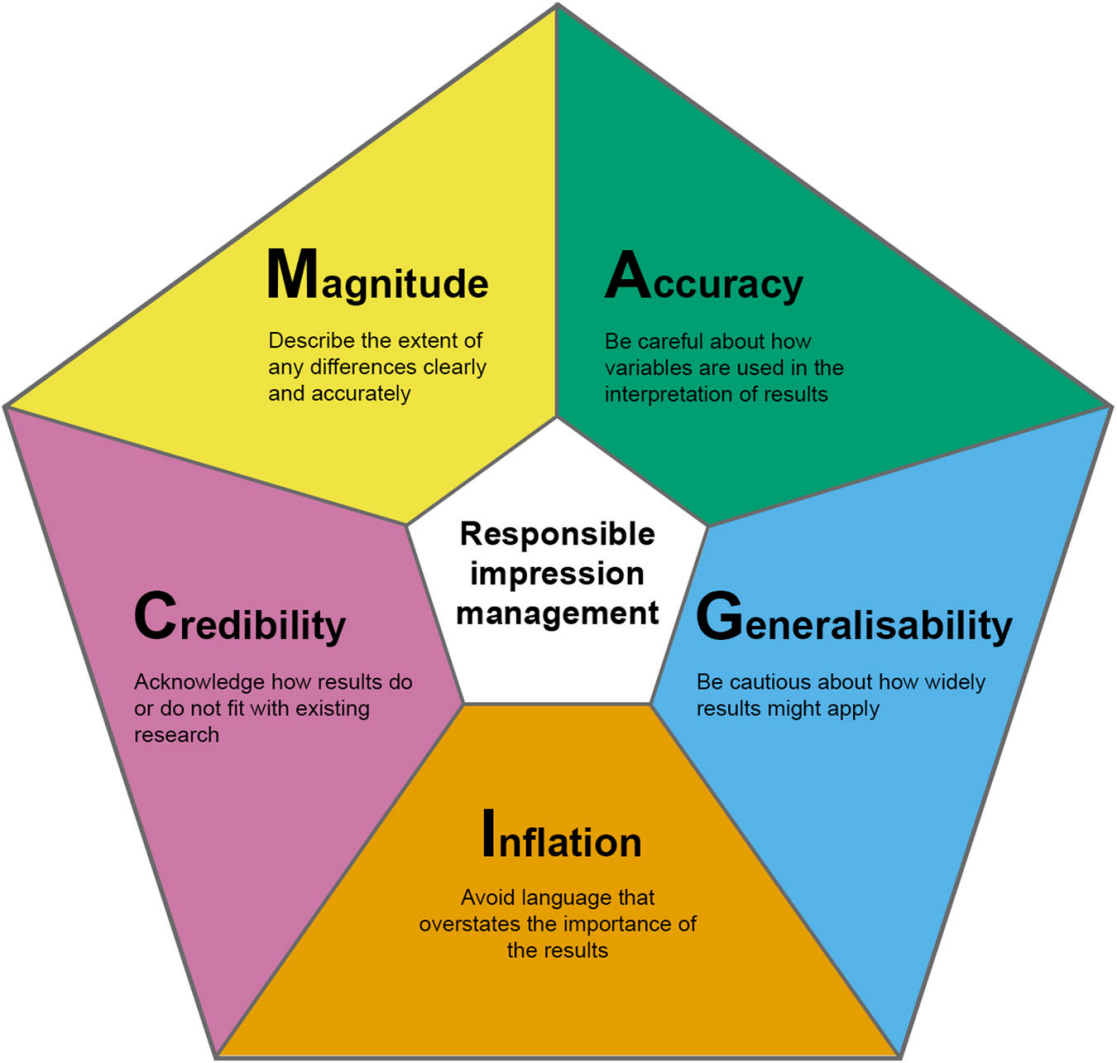
A Touch of Magic in sex/gender neuroscience research reporting: five tests to ensure accurate impression management.

However, less attention has been paid to ensuring linguistic accuracy, of findings ways to avoid problems of overstatement or spin in the narrative language that researchers use to interpret and explain their findings. This has been shown to be a problem in the area of published peer-reviewed sex and gender neuroscience papers^8^. To address this issue, we undertook a consultation exercise to gather feedback on the issues that these criteria could most usefully cover, through workshops with specialist science and science writing groups, public events, and an online survey^15^. As a result of such consultations, we developed a set of five tests for communicating responsibly about sex and gender neuroscience, adapted from Robert P. Abelson’s 5-factor MAGIC framework for organising a principled argument from quantitative evidence (Fig. 1)^16^. We use the term sex/gender in these tests to recognise that in human neuroscience it is difficult to disentangle the influence of participants’ sex - in the sense of biological attributes such as chromosomes and hormones – from the influence of gender - in the sense of socially constructed norms, expectations and experiences that affect patterns of behaviour.

Our intention is that these could augment existing guidelines, to help authors and reviewers avoid common pitfalls in this important area of research, and alert press officers, journalists, science writers and other interested parties to potential problems.

### Magnitude: is the extent of any differences clearly and accurately described?

Authors should state the ratio of statistically significant to non-significant comparisons, in order to draw attention to, for example, the percentage of areas where differences were not found as well as where they were.

Authors should clearly indicate the extent of overlap between female and male data, for example by reporting effect sizes, to avoid giving the impression that a sex/gender difference refers to something that distinguishes all or most of the female cohort from all or most of the male cohort (unless that is what is found).

### Accuracy: are variables clearly defined and carefully used in the interpretation of results?

Authors should make clear how they account for the biological, social and cultural factors associated with sex/gender in interpreting their results.

Authors should apply appropriate caution when interpreting their data in terms of measures that did not form part of the study. For example, when describing the possible causes or behavioural consequences of any average differences in brain imaging data, they should be careful only to refer to brain-behaviour links with a well-established evidence base, beyond stereotypical beliefs.

### Generalisability—are authors cautious about how widely the results might be applied?

Authors should be cautious about the use of phrases such as “women are…” or “men are…”, even where moderated by the term”on average”.

Authors should include appropriate caveats about whether or not their results are likely to apply beyond the study’s demographics, in terms of factors such as age, level of education, occupation, socio-economic status, ethnicity, gender variance, and/or neurodiversity.

### Inflation: do the authors avoid language that overstates the importance of their results?

Authors should ensure they match the strength of their language to the strength of their evidence, avoiding, for example, the unjustified use of terms such as fundamental or profound.

Authors should ensure any discussion of their results accurately reflects the true extent of any differences found.

### Credibility: are authors careful to acknowledge how their findings do or do not fit with existing research?

Authors should acknowledge whether their analytical intent is exploratory or confirmatory, clearly identifying the lower levels of credibility in exploratory studies.

Authors should report the potential limitations of their methodology and analyses, and ensure this has sufficient prominence, including, where appropriate, in the abstract.

## Conclusion

Sex and gender neuroscience research has many key questions to answer, and has the potential for widespread application in many fields. Such research is obviously to be encouraged and supported. However, is worth stressing that, perhaps because of such extensive real-life relevance, it is important that any ambiguities or over-statements are avoided at source, and that safeguards are in place to ensure full transparency and appropriate caution within relevant research reports. Misunderstanding or misinterpretation of such research could have negative real-life consequences, for example by supporting stereotypical beliefs of group inferiority or superiority, or for undermining important social and political initiatives, as well as those concerning physical and mental health.

Over-statement and misrepresentation of research, in the media and beyond, is not, of course, specific to sex and gender research. However, given the extensive general and non-specialist interest in this field, it is an area where published outputs are more likely to be communicated by, and to, non-experts in the field, so we believe an extra layer of caution is appropriate. We suggest that attention to the five impression management factors identified here will support this aim.
